# T-shaped pars plana scleral incision to remove large intraocular foreign body

**DOI:** 10.3389/fmed.2024.1399321

**Published:** 2024-05-14

**Authors:** Mario Damiano Toro, Katarzyna Nowomiejska, Marina Concilio, Lorenzo Motta, Krzysztof Marek Rekas, Ciro Costagliola, Teresio Avitabile, Niccolò Castellino, Georges Caputo, Tomasz Choragiewicz, Robert Rejdak

**Affiliations:** ^1^Eye Clinic, Public Health Department, University of Naples Federico II, Naples, Italy; ^2^Department of General and Pediatric Ophthalmology, Medical University of Lublin, Lublin, Poland; ^3^Department of Medicine and Health Sciences "V. Tiberio", University of Molise, Campobasso, Italy; ^4^Department of Ophthalmology, William Harvey Hospital, East Kent Hospitals University NHS Foundation Trust, Ashford, United Kingdom; ^5^Students’ Scientific Association at the Chair and Department of General and Pediatric Ophthalmology, Medical University of Lublin, Lublin, Poland; ^6^Eye Clinic, Department of Neuroscience, Reproductive Sciences and Dentistry, University of Naples Federico II, Naples, Italy; ^7^Department of Ophthalmology, University of Catania, Catania, Italy; ^8^Department of Pediatric Ophthalmology, Rothschild Foundation Hospital, Paris-Cedex, France; ^9^Chair and Department of General and Pediatric Ophthalmology, Medical University of Lublin, Lublin, Poland

**Keywords:** intraocular foreign body, ocular trauma, pars plana vitrectomy, scleral wound, surgical techinque

## Abstract

**Aim:**

To investigate the efficacy and safety profile of T-shaped pars plana scleral incision technique in removing large intraocular foreign bodies, during 23-gauge pars plana vitrectomy.

**Methods:**

Retrospective interventional case series that included patients diagnosed with a large intraocular foreign body (IOFB). Possible postoperative complications were recorded 24 h, 1 month, 3 and 6 months postoperatively.

**Results:**

Thirty eyes of 30 patients (48 ± 5 years old) were enrolled. All IOFBs were successfully removed: mean diameters of 7.8 ± 2.0 mm and 2.6 ± 0.3 mm. Silicone oil and sulfur hexafluoride were used in 27 and 3 eyes, respectively. Lensectomy was performed in 27 eyes. Intraocular lens was implanted at first attempt in 12 eyes; during a second operation in 12 eyes and 6 eyes remained aphakic. At any follow-up, no signs of postoperative complications were observed. Secondary retinal detachment occurred in 12 eyes. Mean preoperative corrected distance visual acuity was 0.04, on the Snellen scale; it increased to 0.07, at last follow-up. Mean intraocular pressure was 17.97 mmHg. All eyes were preserved.

**Conclusion:**

T-shaped scleral incision could be an effective, safe and easy-to-perform standard procedure to remove large IOFBs during pars plana vitrectomy, without increasing the risk of surgical complications and additional damage to the ocular tissues.

## 1 Introduction

Ocular trauma represents one of the most sight-threatening eye conditions whose clinical spectrum is extremely variable according to injury mechanisms and structural eye damage (1–4). Ocular trauma associated with intraocular foreign bodies (IOFBs) is a serious ocular condition that may lead to disastrous consequences, such as toxic effects, chronic inflammation, development of fibrocellular proliferation, retinal traction, and possible detachment, endophthalmitis, or phthisis bulbi and permanent visual loss (5, 6).

The management of posterior segment IOFBs is challenging due to the complex presentation and several goals need to be achieved during the treatment, such as foreign body removal, ocular integrity preservation, and functional restoration (5). Currently, pars plana vitrectomy (PPV) is the gold-standard procedure for diseases affecting the posterior eye segment including retained IOFBs (7, 8). Indeed, it enables the surgeon to directly visualize the foreign body and to perform IOFB-controlled removal, reducing surgical complications and improving postoperative outcomes (9, 10). Recently, sutureless vitrectomy systems have been developed, becoming more frequently used in complicated vitreoretinal disease treatment (10–12). The sutureless systems minimize surgically-induced trauma to the conjunctiva and sclerotomy sites, improving operative efficiency and hastening postoperative recovery (8). Additionally, the decrease of conjunctival scarring may result in higher success rates allowing surgeons to attempt additional conjunctival-scleral surgical procedures (13, 14).

To the best of our knowledge, despite the advances in microsurgical and vitreoretinal techniques, there is no established consensus regarding the best-standardized technique to remove a large IOFB retained in the eye posterior segment. Besides reaching the primary goal of the operation – IOFB removal - special care must be devoted to reducing iatrogenic lesions (15, 16). Indeed, due to its size and incarceration, an IOFB extraction through its entry could potentially cause serious iatrogenic lesions to intraocular structures, especially when the size of the wound is far smaller than the maximal diameter of the IOFB, due to its rotation and tumbling (15). Standard visualization techniques cannot often precisely identify the IOFB’s size and location (17) and the routinely applied surgical techniques are often inadequate in cases of large IOFBs (18) and additional sclerotomies need to be performed. Moreover, the scleral incision occasionally needs to be enlarged to extract the IOFB, performing a surgical incision/cut directed parallelly to the corneal limbus (19). Despite long incision allowing an easier IOFB extraction, it could increase intraoperative risks, such as bleeding, intraocular pressure instability, poor visualization during manipulation, and postoperative complications, in terms of wound leakage and high astigmatism.

To reduce the length of incisions and the associated complication rate, we propose a modified scleral approach in which an additional vertical scleral incision (T-shaped sclerotomy) is performed, which may provide a comparable manipulation area with shorter incisions. We aim here to describe this new technique of scleral incision for extraction of IOFBs, which can enhance the potential size of IOFBs removed without an increase in possible iatrogenic lesions or complications, and to evaluate the anatomic and functional outcomes as well as the safety profile of the technique.

## 2 Materials and methods

### 2.1 Study design

This was a retrospective, single-arm, interventional study. A consecutive series of patients, who were diagnosed with open globe injury [according to Birmingham Eye Trauma Terminology (20)] and underwent 23-gauge (G) vitrectomy surgery with T-shaped pars plana scleral incision technique to remove large IOFBs within 24 h from emergency unit admission, at the Department of General Ophthalmology of the University of Lublin, between January 2015 and July 2020, were enrolled. The study protocol conformed to the principles of the Declaration of Helsinki and was approved by the Local Research Ethics Committee (number: KE−0254/342/2018). A large IOFB was defined as an intraocular object whose major size was bigger than 5 mm. We excluded all eyes that had previous ocular surgery, previous penetrating injury, amblyopia, scleromalacia, or any ocular condition that could negatively interfere with the final outcomes. Pre-, intra-, and postoperative data were collected.

### 2.2 Examination

At baseline, we recorded the following data: patients’ demographic, history of pre-existing systemic and local diseases, history of the injury, the timing between the trauma and hospital access, corrected distance visual acuity (CDVA) measured with Snellen decimal scale, intraocular pressure (IOP), slit lamp biomicroscopy, fundus examination, entry site of the IOFB and location, lesions of ocular structures. For CDVA analysis, counting fingers, hand movement and no light perception were converted into Snellen decimal values (21). The presence of IOFB in the posterior segment was confirmed by preoperative B-scan ultrasonography and/or computed tomography (CT) examination of the orbit and the globe. Systemic and topical antibiotics were administered to all patients.

Microtome was used to measure all IOFBs after surgical extraction. Postoperative data were collected 24 h and 6 months after the surgery and included: CDVA, IOP, biomicroscopic anterior segment examination, including Seidel test, and dilated fundus oculi. IOP was measured by the gold standard technique of Goldman applanation tonometry and calculated as the mean value of three consecutive measurements. Hypotony was defined as an IOP equal to or inferior to 5 mmHg. At each examination, possible early and delayed complications in terms of vitreous hemorrhage, choroidal hemorrhages, retinal detachment, clinical signs of hypotony (such as Descemet folds, choroidal and retinal folds), and hypotony-related complications (ciliochoroidal detachment, suprachoroidal detachment, collapsed eye) and endophthalmitis were evaluated and recorded.

### 2.3 Surgical technique

**Figure 1:**
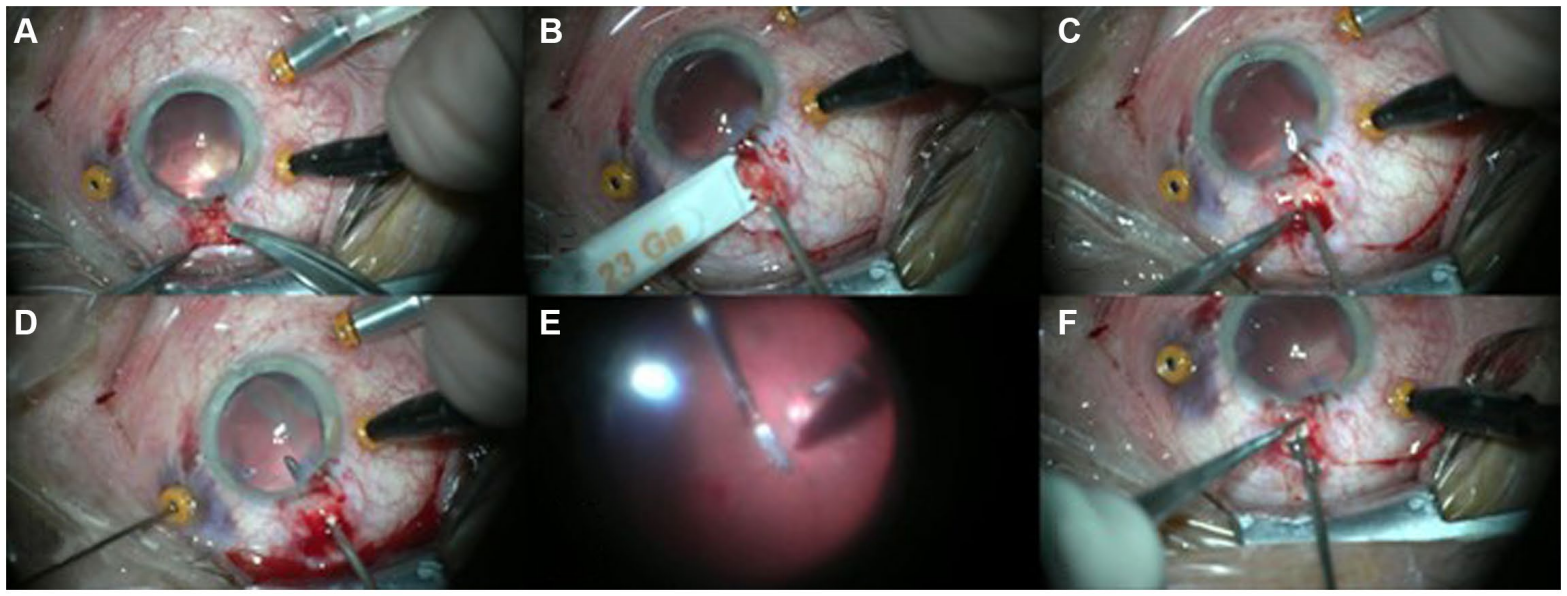
T-shaped pars plana scleral incision steps: **(A)** Preparation of conjunctiva to expose sclera at 12 o’clock position. A chandelier light is installed on the microcannula. **(B)** At 4 mm from limbus, a parallel cut is created with the blade of the trocar knife. **(C)** In the middle of the scleral incision, an additional perpendicular cut is created (T-shaped incision). **(D)** Intraocular foreign body (IOFB) is bimanually elevated from the posterior pole with two forceps: 23-gauge (g) installed through microcannula, 20 g through sclera incision. **(E,F)** IOFB is removed from the eye with 20G forceps through the T-shaped incision.

All patients underwent 3-port, 23-gauge (G) standard PPV under general or retrobulbar anesthesia, according to the patient’s health condition, after that the surgeon have closed the primary penetrating open globe wound. All operations were performed at one hospital by the same surgeon (R.R.) with the Constellation Vision System (Alcon Laboratories, Inc., Fort Worth, TX) using the Edgeplus trocar system (Alcon Laboratories, Inc), a conventional solid shaft-type trocar–cannula system. Wide-angle fundus visualization was achieved by using the microscope-mounted noncontact wide-field imaging system (BIOM, Oculus, Munich, Germany). The operative eye was prepped and draped in the usual sterile ophthalmic fashion with 5% povidone-iodine. All phakic patients underwent a clear cornea lens extraction via phacoemulsification, and if capsular support was preserved an intraocular lens (IOL) was placed in the bag or sulcus. A standard three-port 23G pars plana vitrectomy with peripheral shaving of the vitreous base was performed using triamcinolone as a visual aid. After the vitrectomy was completed, 1 cm^3^ of perfluorocarbon liquid (PFCL) was injected to cover the posterior pole and protect the macula from injury from a falling IOFB (14). Conjunctiva was displaced behind the limbus (Figure 1A). Then, A separate T-shaped scleral incision was performed with a blade of a trocar knife (Supplementary Digital Content S1) as follows: (1) incision, with the length depending on IOFB size, at 4.00 mm and parallel to the limbus; (2) additional 1.5 mm perpendicular radial incision, in the middle of the previous cut, toward the limbus (Figure 1C). All main surgical steps were displayed in Figure 1.

The most appropriate site of incision was chosen after computed tomography (CT) visualization, during the intraoperative examination, considering the size of IOFB, its localization, and positions of sclerotomies. In standard cases with floating IOFB, the preferable localization of incision was 12 o’clock. Intraocular foreign body (IOFB) was bimanually elevated from the posterior pole with two forceps: 23G Grieshaber Maxgrip forceps (Alcon Laboratories, Inc., Fort Worth, TX) installed through microcannula and a 20G Grieshaber Maxgrip forceps (Alcon Laboratories, Inc., Fort Worth, TX) through the T-shaped scleral incision. After IOFB removal, a few drops of triamcinolone acetonide were used to mark potential vitreous strings coming out from sclerotomy, which were removed with the vitrectomy cutter. The T-shaped scleral incision was sutured with absorbable stitches (Vicryl 7.0, Johnson and Johnson USA). Fluid-air exchange was performed with subretinal fluid drainage, in case of retinal detachment, with the 23G PPV probe and refined with a 23G Backflush Soft Tip (Alcon Laboratories, Inc., Fort Worth, TX). PFCL fluid was aspirated. Laser was applied to coagulate all retinal lesions and peripheral retina, bordering the pars plana scleral incision. Then, non-expanding sulfur hexafluoride (SF6 20%) gas or silicone oil was applied as a tamponade agent. At the end of the procedure, leakages of standard sclerotomies, that did not self-seal after three 20-s sessions of pressure to the incision with a cotton tip applicator, were sutured with additional absorbable stitches. For all the patients, topical treatments were prescribed as follows: prednisolone acetate in tapering fashion, atropine 1%, and moxifloxacin drops 5 times/day for 4 weeks.

### 2.4 Statistics

The values of CDVA and IOP were expressed as a mean value ± standard deviation (SD). Descriptive statistics of all complications, before and after the surgical procedure, were collected. The mean CDVA and IOPs after treatment were compared by analysis of variance; if significant, multiple comparisons were performed with the Tukey–Kramer test. Spearman’s rank correlation coefficient was used to evaluate the relationship between factors such as the age of the patient and the largest diameter of IOFB with IOP and CDVA. Wilcoxon’s test was used to evaluate the visual results (CDVA) 6 months after the surgery compared to the pre-operative CDVA. Friedman’s ANOVA was used to assess differences in IOP in the pre-operative state, at 1 day and 6 months after the surgery. *p* values below 0.05 were considered statistically significant. Statistical analyses used SPSS for Windows, version 16.0 (SPSS, Inc., Chicago, IL) and Statistica 13 software (StatSoft, USA).

## 3 Results

Thirty cases (27 Caucasian and 3 Afro-American patients) met the inclusion criteria (26 men and 4 women) with a mean age of 48 ± 5 years,. All IOFBs were metal (one with an additional rubber element) with an average length of 7.8 ± 2.00 mm and a width of 2.6 ± 0.3 mm. The mean preoperative CDVA was 0.034 ± 0.011. Three eyes were primarily aphakic. In 27 eyes the crystalline lens was removed; in 12 cases the intraocular lens was implanted in a preserved bag or in the sulcus at the first attempt – in case of posterior capsule rupture, while in 12 eyes the lens was implanted during a second surgery and finally 6 eyes remained aphakic. Intraocular tamponades were used: silicone oil in 27 eyes, and non-expanding SF6 20% gas in 3 cases. Secondary retinal detachment due to PVR occurred in 12 eyes. Dissection of the PVR was performed and silicone oil was used as a tamponade to achieve retinal reattachment.

**Figure 2:**
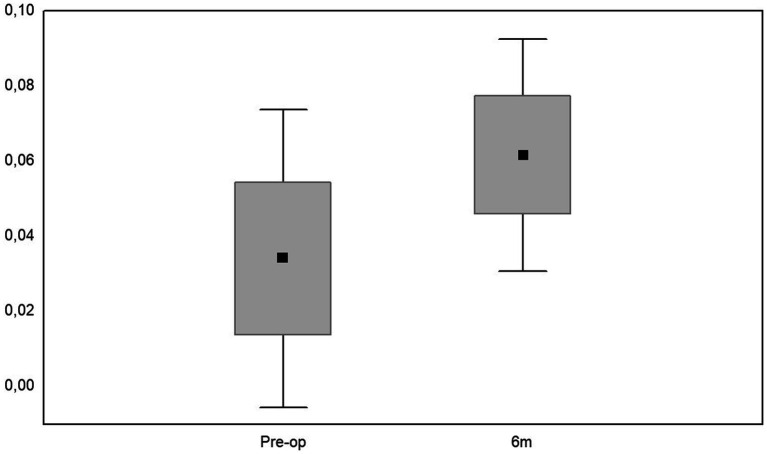
Mean values, median numbers, standard deviations, and ranges of corrected distance visual acuity (CDVA), preoperatively and at 6 months of follow-up.

**Figure 3:**
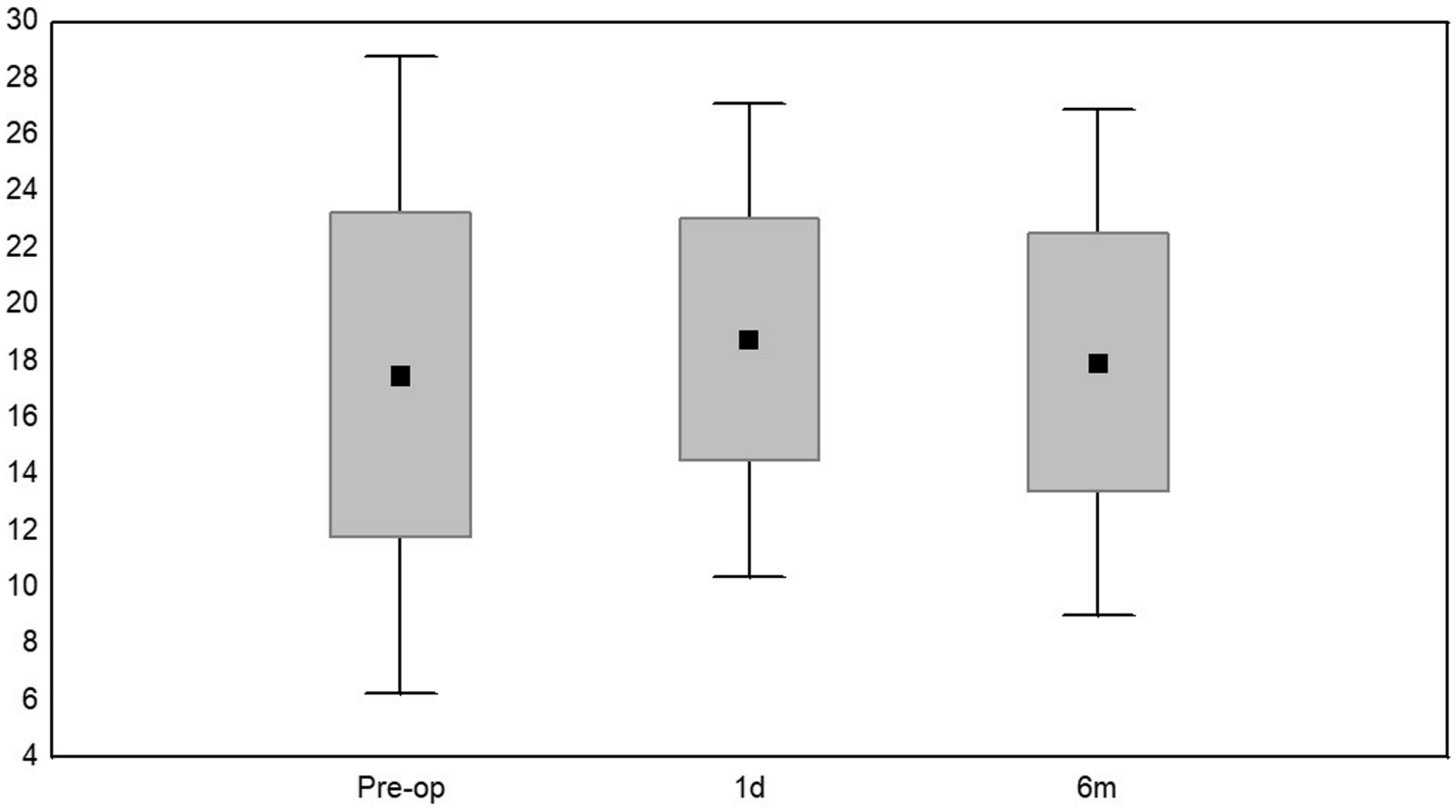
Mean values, median numbers, standard deviations, and ranges of intraocular pressure (IOP): preoperatively, at 24 h and 6 months of follow-up.

At the final follow-up, retina was reattached in all the eyes, mean CDVA was 0.06 ± 0.08. Compared to preoperative data, CDVA improved in 13 cases (43.3%), was stable in 14 eyes (46.7%) and worsened in 3 eyes (10%) (Figure 2; Table 1). Spearman’s rank correlation coefficient analysis revealed that the largest diameter of IOFB (length) did not influence the visual outcome at 6 months follow-up and did not correlate with intraocular pressure, neither in the pre-operative state nor in postoperative visits (1 day, 6 months after the surgery) (Table 2). At last follow-up, the mean IOP was 17.97 ± 4.55 mmHg (Figure 3; Table 3). Increased IOP was controlled with topical treatment in 7 eyes filled with silicone oil. During follow-up, no eyes showed signs of hypotony, choroidal folds, uveal prolapse, oil or fluid leakage through the sclera incision, intravitreal or suprachoroidal bleeding. No eyes showed signs of endophthalmitis, atrophy and required enucleation.

**Table 1:** Mean values, median numbers, standard deviations, and ranges of corrected distance visual acuity (CDVA).

Time	Mean value (± SD)	Me (Q_1_,Q_2_)	Range
Pre-op	0.034 ± 0.112	0.004 (0.003–0.01)	0.000–0.600
6th month	0.062 ± 0.086	0.05 (0.004–0.05)	0.000–0.400
*P* ^*^	0.026		

*Wilcoxon’s test; Me, median number; SD, standard deviation; Q_1_,Q_2_, quartile.

**Table 2:** Results of Spearman’s rank correlation coefficient analysis between IOFB length and CDVA at baseline and 6 months postoperatively; and between IOFB lenght and IOP at baseline, 1 day and 6 months postoperatively.

**IOFB Length correlation with**	**R Spearman**	*p*	
Pre-op CDVA	-0.25	0.19	
6th month CDVA	-0.32	0.09	
Pre-op IOP	0.06	0.78	
1st day IOP	-0.04	0.86	
6th month IOP	0.20	0.32	

CDVA, corrected distance visual acuity; IOP, intraocular pressure number; Pre-op, preoperative; IOFB, intraocular foreign body.

**Table 3:** Mean values, median numbers, standard deviations, and ranges of intraocular pressure (mmHg).

Time	Mean value (SD)	Me (Q_1_,Q_2_)	Range
Pre-op	17.52 ± 5.74	15.0 (13.0–21.0)	10.0–31.0
1st day	18.76 ± 4.27	17.0 (16.0–22.0)	12.0–27.0
6th month	17.97 ± 4.55	18.0 (15.0–20.0)	7.0–30.0
P*	0.272		

*Friedman’s ANOVA; Me, median number; SD, standard deviation; Q_1_,Q_2_, quartile; Pre-op, Preoperative.

## 4 Discussion

Ocular trauma is the most common preventable cause of unilateral visual impairment. It mostly affects young men, in the working-age population (22). Our data show a similar distribution to the epidemiological data reported in the literature; indeed, in our sample, males were predominant (86.7%) and underwent eye trauma at the workplace. Despite European epidemiological data not being available, it is estimated that approximately 1.6 million people are blind due to ocular injuries, worldwide (23). Ocular injury substantially impacts the quality of life, causing both psychological and physical stress (24).

The spectrum of globe injuries is extremely variable, ranging from isolated superficial cuts to complex severe trauma, involving many ocular structures. Therefore, the visual prognosis may deeply vary. Among the ocular injuries, IOFBs still represent the major cause of morbidity in young people. Commonly, most of the IOFBs are metallic, but several different materials such as wood, glass, stone, and plastic may often cause trauma (25). In our study group, the injuries involved one eye and were related to the presence of a single metallic object, as well.

In diagnosing IOFBs, orbital imaging is crucial: computed tomography is the first choice to find and locate IOFBs; whereas magnetic resonance imaging (MRI) should be avoided until metal foreign bodies are excluded (26).

Nowadays, modern intraocular surgical techniques have radically changed the outcome of patients who underwent ocular trauma allowing vision to be restored even in hopeless cases. It is crucial to accurately diagnose open globe injury (OGI) and evaluate several features of possible IOFBs, such as the size, length, volume, and mass (27–29). Many authors suggested a positive correlation between the weight, the dimension of the IOFB, and complications such as hyphema, vitreous or retinal hemorrhages, and uveal prolapse (27). Nevertheless, the presence of IOFB is not strictly related to functional outcomes. Furthermore, many factors need to be considered: ocular damages related to the injury, possible iatrogenic injuries during surgical procedures, and postoperative complications.

Before the development and diffusion of PPV as a standard technique, all the magnetic IOFBs were extracted from the posterior segment through a scleral incision with an external magnet, with possible intra- and post-operative complications. Indeed, surgeons performed an uncontrolled removal of the IOFB, embedded in the retina or in the vitreous bands, that could lead to vitreous hemorrhages, proliferative vitreoretinopathy (PVR), and high risk of retinal detachment (RD), in up to 43% of cases (30). Nowadays, PPV allows surgeons to perform the whole procedure under direct visualization (30), to protect the macular area using PFCL during metallic IOFB removal (31) and to lower the number of iatrogenic damages (32). Moreover, PPV allows for the use of silicone oil, which is related to improved post-operative visual acuity (33). Although PPV brought novelty and safety to IOFB extrapolation, nowadays there is no consensus on a standard technique (28).

Sborgia et al. have shown that sutureless 25-gauge pars plana vitrectomy may be useful in the removal of IOFBs because it is associated with a less traumatic appearance, less conjunctival damage, less intraocular inflammation, and faster healing of sclerotomies compared with 20-gauge PPV (34). To our knowledge, there is no data comparing the 25-gauge to the 23-gauge system. In all our cases a 23G PPV was used.

The technique of removal of IOFBs is closely related to their size and volume: large IOFBs often require extraordinary measures with routine changes in the surgical plan (18), while small and medium-sized IOFBs can be extracted directly from the traumatic access wound, which can be from both the sclera and the anterior segment, usually through a corneal incision. Several authors have described their own technique for removing IOFBs from the anterior segment.

Lin Z et al. described the “intraocular lens blocking technique”: they performed phacoemulsification, posterior capsulotomy and insertion of a three-piece intraocular lens (IOL), and then lifted the IOFBs from the posterior segment through the capsule opening and the IOL edge into the anterior chamber, using forceps and the IOL to prevent the IOFBs from slipping onto the retina. Finally, the IOFBs were removed through the main incision in the cornea (35). The most important aspect to consider when removing IOFBs from the corneal incision is the postoperative corneal astigmatism and, of course, the progressive loss of endothelial cells (36). To avoid these complications, some surgeons have suggested limbus-parallel or scleral approaches. Dhoble P and colleagues reported on a case series of 14 patients in whom the posterior IOFBs were removed through a posterior capsular defect and a triplanar sclerocorneal tunnel using a magnet. According to their results, there was a minor complication of IOFB slippage during extraction compared to the use of forceps (37). Grabbing the IOFB inside the posterior segment and pulling it out through the sclera incision, represents one of the most challenging surgical steps. Recently, Li H. et al. compared the use of magnet and forceps in removing metallic posterior IOFBs; they highlighted the efficacy and efficiency of magnetic bar in reducing the possibility of IOFB slippage and fall, preventing iatrogenic retinal damage, and shortening the time taken to its removal (38).

Beyond the type of instrument used to grab the IOFBs, inappropriate incision size can cause IOFB to fall on the retina, representing a potential source of iatrogenic lesions (39). Therefore, it is mandatory to adequately adjust the cut construction, size and shape of the scleral wound to ease the surgical maneuvers according to the IOFB size. In case of scleral incision, length can be shortened by creating an additional perpendicular incision as previously demonstrated by L-shaped scleral incision adoption for scleral IOL implant and extraction which allowed a corneal astigmatism reduction. While scleral scar tissue tends to contract along the horizontal axis, perpendicular incision seems to be neutral for the astigmatism (40). Furthermore, the more peripheral the horizontal cut is localized, the less corneal distortion is generated. Accordingly, the optimal T-shaped scleral horizontal cut should be created in the most peripheral zone of the pars plana whereas the perpendicular cut length should be limited by the length of pars plana, approximately at 1.5 and 4 mm from the limbus in eyes with “normal” axial length, respectively. In the previously described technique, the incision position and size varied according to the axial length of the eye and lens status (41). In our study, to reduce risk of retinal detachment and infection, the wound has been carefully cleaned from incarcerated vitreous strings, the scleral and conjunctival wounds sutured with absorbable stitches. No specific complication at the location of the T-shaped sclerotomy was observed in our series.

Regarding the technique, to date, no studies have considered the potential impact of combined primary cataract extraction and PPV on functional outcomes. The combined surgery is influenced by several factors, such as the presence or absence of a traumatic cataract, damage to the capsular bag or zonular supports (36). As described by Castaldelli et al. (42), both scleral and iris fixation can be performed, depending on the characteristics of the eye and the experience of the surgeons.

In our cases, in only 12 cases the IOL was implanted in a preserved bag or in the sulcus during the primary surgery. Out of 30 eyes, 12 eyes were implanted during a second surgery and 6 eyes remained aphakic.

Our results are not consistent with previous studies in which larger IOFB size was one of the main influencing factors for poor visual recovery (28, 29). In these cases, large IOFBs were associated with larger incisions (43), resulting in higher aqueous flow, lower visibility, and stability of ocular field pressure during surgery, and increased risk of complications such as wound bleeding, leakage, conjunctival blistering, choroidal effusion, fibrous ingrowth, fibroplasia, anterior segment ischemia, and corneal astigmatism (44). In our cases, the only side effect observed was increased intraocular pressure, which was controlled with topical treatment in 7 eyes filled with silicone oil. No further complications were noted during follow-up. Therefore, according to our findings, the T-shaped pars plana incision seems to be a promising and safe technique for the removal of large IOFBs.

The study has some limitations: for instance, the retrospective design, small sample size and the short period of follow-up. Therefore, further studies with longer period of follow-up and a larger number of patients are highly recommended to confirm our findings. An interesting aspect that could be investigated in future studies are the differences in terms of possible intraoperative and postoperative complications (both early and late), anatomical and functional outcomes between combined primary cataract extraction and PPV and secondary IOL implantation.

## 5 Conclusion

Ocular trauma with IOFB represents one of the primary causes of blindness in young men. However, there is still no consensus regarding a standardized technique to perform IOFB removal. The novel technique for scleral incision with a specific incision design and two cuts can reduce incision cuts’ lengths and increase the extraction area. In addition, it represents a safe technique as neither iatrogenic lesions nor increased intra- and/or post-surgical complications were recorded. Therefore, the T-shaped scleral incision seems an effective and easy technique, to apply during PPV, for large and incarcerated IOFB removal from the posterior segment.

## Data availability statement

The raw data supporting the conclusions of this article will be made available by the authors, without undue reservation.

## Ethics statement

The studies involving humans were approved by the Local Research Ethics Committee, University of Lublin (number: KE−0254/342/2018). The studies were conducted in accordance with the local legislation and institutional requirements. The participants provided their written informed consent to participate in this study.

## Author contributions

MDT: Conceptualization, Formal analysis, Methodology, Writing – review & editing. KN: Conceptualization, Writing – original draft. MC: Writing – review & editing. LM: Writing – original draft. KMR: Investigation, Writing – original draft. CC: Supervision, Writing – original draft. TA: Formal analysis, Writing – original draft. NC: Formal analysis, Writing – original draft. GC: Supervision, Writing – original draft. TC: Investigation, Methodology, Writing – original draft. RR: Supervision, Writing – original draft.
